# Prognostic Features for Overall Survival in Male Diabetic Patients Undergoing Hemodialysis Using Elastic Net Penalized Cox Regression; A Machine Learning Approach

**DOI:** 10.34172/aim.27746

**Published:** 2025-01-01

**Authors:** Mehrdad Sharifi, Razieh Sadat Mousavi-Roknabadi, Vahid Ebrahimi, Robab Sadegh, Afsaneh Dehbozorgi, Seyed Rouhollah Hosseini-Marvast, Mojtaba Mokdad

**Affiliations:** ^1^Emergency Medicine Department, School of Medicine, Shiraz University of Medical Sciences, Shiraz, Iran Emergency Medicine Research Center, Shiraz University of Medical Sciences, Shiraz, Iran; ^2^Health System Research, Vice-Chancellor of Treatment, Shiraz University of Medical Sciences, Shiraz, Iran Department of Biostatistics, School of Medicine, Shiraz University of Medical Sciences, Shiraz, Iran; ^3^Department of Community Medicine, School of Medicine Shiraz University of Medical Sciences, Shiraz, Iran; ^4^Allergy Research Center, Shiraz University of Medical Sciences, Shiraz, Iran; ^5^Gomel State Medical University, Gomel, Belarus

**Keywords:** Diabetic, Elastic net, Hemodialysis, Machine learning, Male

## Abstract

**Background:**

Diabetics constitute a significant percentage of hemodialysis (HD) patients with higher mortality, especially among male patients. A machine learning algorithm was used to optimize the prediction of time to death in male diabetic hemodialysis (MDHD) patients.

**Methods:**

This multicenter retrospective study was conducted on adult MDHD patients (2011-2019) from 34 HD centers affiliated with Shiraz University of Medical Sciences. As a special type of machine learning approach, an elastic net penalized Cox proportional hazards (EN-Cox) regression was used to optimize a predictive regression model of time to death. To maximize the generalizability and simplicity of the final model, the backward elimination method was used to reduce the estimated predictive model to its core covariates.

**Results:**

Out of 442 patients, 308 eligible cases were used in the final analysis. Their death proportion was estimated to be 28.2%. The estimated overall one-, two-, three-, and eight-year survival rates were 87.6%, 74.4%, 67.2%, and 53.9%, respectively. The EN-Cox regression model retained 14 (out of 35) candidate predictors of death. Five variables were excluded through backward elimination technique in the next step. Only 6 of the remaining 9 variables were statistically significant at the level of 5%. Body mass index (BMI)<25 kg/m^2^ (HR=2.75, *P*<0.001), vascular access type (HR=2.60, *P*<0.001), systolic blood pressure (1.02, *P*=0.003), hemoglobin (11≤Hb≤12.5 g/dL: HR=3.00, *P*=0.028 and Hb<11 g/dL: HR=2.95, *P*=0.021), dialysis duration in each session≥4hour (HR=2.95, *P*<0.001), and serum high-density lipoprotein cholesterol (HDL-C) (HR=1.02, *P*=0.022) had significant effects on the overall survival (OS) time.

**Conclusion:**

Anemia, hypotension, hyperkalemia, having central venous catheter (CVC) as vascular access, a longer dialysis duration in each session, lower BMI and HDL-C were associated with lower mortality in MDHD patients.

## Introduction

 Diabetes mellitus (DM) accelerates the reduction of glomerular filtration which leads to the development of end-stage renal disease (ESRD).^1,2^ The administration of renal replacement therapy (RRT), which removes toxic substances and extra fluids, is a life-saving treatment in patients with ESRD.^3^ RRT includes hemodialysis (HD), peritoneal dialysis (PD),^3-5^ and kidney transplantation (KT).^6^ Among RRTs, HD is the most popular treatment, followed by PD and KT.^7^ Previous studies have shown that the survival of patients undergoing HD is affected by several factors some of which are modifiable^8^ including serum albumin concentrations,^9^ body fat tissue,^10^ hemoglobin A1C (HbA1C) levels,^11^ serum lipids,^12^ serum ferritin levels,^13^ and white blood cell (WBC) and red blood cell (RBC) counts.^14^

 Gender has been acknowledged as one of the strongest prognostic factors for survival of patients undergoing HD. The progression of chronic kidney disease (CKD) is different in genders due to various factors. Several researches have reported that the male gender (especially men with diabetic nephropathy) is at higher risk of progression to ESRD and death.^15,16^ Although the exact biological mechanisms for the faster decrease of the estimated glomerular filtration rate (eGFR) in men are unknown, previous studies have suggested various theories.^17^ For instance, an unhealthier lifestyle, the detrimental effects of testosterone, lack of estrogen’s protective effects in men, sex differences in the metabolism of nitrogen oxide, oxidative stress, and the actions of sex steroids can be mentioned in this regard.^15-19^

 In the study of survival time data, larger sample sizes and more events of interest are frequently preferred. Previous simulation studies have shown that fitting survival models with multiple covariates using ordinary regression procedures with small sample data may lead to bias in the estimation of the coefficients since the outcome events per candidate covariate (OEPCC) are too few. This can lead to unstable predictions and the regression models may perform poorly on new datasets.^20-22^ Among the different techniques to model the survival time data, the Cox proportional hazards (PH) regression model is the best known procedure because it has fewer assumptions.^23,24^ As a rule of thumb, a minimum of five to twenty OEPCC are needed for reliable results in the Cox PH model.^20-22^ However, when the sample size is relatively small, if the number of the candidate covariates is relatively large, the number of the OEPCC tends to be smaller than expected and using ordinary survival time methods can be misleading.^21,22^ In this situation, using the elastic net penalized Cox (EN-Cox) regression model, as a special type of machine learning approach, is the best option.^22,25^ The elastic net regressions solve this problem by adding a penalty term to the log-likelihood function (including the unknown parameters of the model) which is used to estimate the model parameters.

 In general, narrowing down a large set of covariates to a smaller one can improve our understanding of the most significant predictors of death. To select the variables, machine learning procedures such as the elastic net and the least absolute shrinkage and selection operator (LASSO) regressions can be used.^25^

 Diabetic patients constitute a significant percentage of HD patients and have higher mortality, especially male patients,^15,16^ than nondiabetic patients.^1^ Therefore, the current study aimed to derive a parsimonious model for predicting overall survival (OS) among male diabetic HD (MDHD) patients and to determine its associated factors. In the present study, as a special type of machine learning method, the EN-Cox regression model (which has not been used for MDHD patients’ data so far) was applied to optimize the prediction of time to death.

## Materials and Methods

###  Study Design and Setting

 This multicenter retrospective cohort study was conducted on all MDHD patients who referred to 34 hospitals affiliated with Shiraz University of Medical Sciences (SUMS) from June 29, 2011 to March 1, 2019. The inclusion criteria were all MDHD patients with the age of ≥ 18 years with at least 4 months since their first HD. Patients with high missing data rates due to unknown last status were excluded from the study. The patients’ demographic characteristics, laboratory and clinical findings, and outcomes were extracted from the Special Diseases Database of SUMS.

###  Statistical Analysis

 The time interval from four months after the first HD to the end of follow-up was considered as the censored time if the desired event (i.e. death) did not happen during it. The patients’ survival probability was estimated using the non-parametric Kaplan-Meier (KM) method and the various groups were compared via the log-rank test.^24^

###  The Elastic Net Penalized Cox Model

 When the sample size is relatively large, the model parameters can be accurately estimated via the conventional maximum likelihood method.^21,25^ In most cases, however, the sample size is not large enough to achieve unique and reliable coefficient estimates. Here, stable results can be generated by using the penalized version of the objective (or log-likelihood) function.^20,25^ As special types of machine learning methods, the LASSO and Ridge models are two different types of penalization techniques that shrink the regression coefficient estimates towards zero to achieve unique estimates.^25,26^ (See Supplementary file 2 for more information) Unlike the Ridge model which always creates a prognostic regression model that includes all the candidate covariates, the LASSO algorithm performs variable selection, as well. In fact, the LASSO technique results in a sparse model, i.e. a regression model that includes only a small subset of the covariates.^26^

 As a machine learning approach, the elastic net penalized model is a combination of the LASSO and Ridge regression models^25,26^ and its log-likelihood function can be formulated as follows:


(1) $$l_{elastic-net}=\log L_{elastic-net}=\log L+\lambda \left(\left(1-w\right)\times ridge\;penalty+w\times LASSO\;penalty\right)$$

Where *log* L is an unpenalized log-likelihood function, while *w* and 𝜆 are regularization parameters which are data-dependent and *a priori* values cannot be attributed to them. The LASSO (*w*= 1) and Ridge (*w*= 0) models are specific cases of the elastic net model.^25,26^

 The main challenge is to specify these regularization parameters for which the cross-validated log-likelihood function of the fitted model is maximum. The 5-fold cross-validation method was applied. To perform cross-validation, the dataset was randomly divided into five equal folds. First, one fold was held out and a separate regression model was trained on all the other folds. Then, the trained model was tested on the held-out fold and the prediction error was calculated. After repeating this process and using all the five folds as the validation sets, the average of the five calculated errors was called the ‘cross-validation error’.^26^

 When the OEPCC are too few, instead of the conventional Cox-adjusted PH model, an alternative penalized regression can be utilized. In this study, an EN-Cox algorithm was used to model time to death in MDHD patients. Similar to the LASSO regression, the elastic-net algorithm performs variable selection by setting some model parameter estimates exactly to zero. The variables selected by the EN-Cox method were then entered into an unpenalized Cox model to specify a baseline for comparison during model development. In order to reduce the number of variables in the baseline model and to obtain a parsimonious one, the backward elimination procedure was used.^25,26^ All the statistical analyses were done using the “*survival*” and “*glmnet*” packages in the R statistical software (version: 3.6.3).

## Results

**Figure 1:**
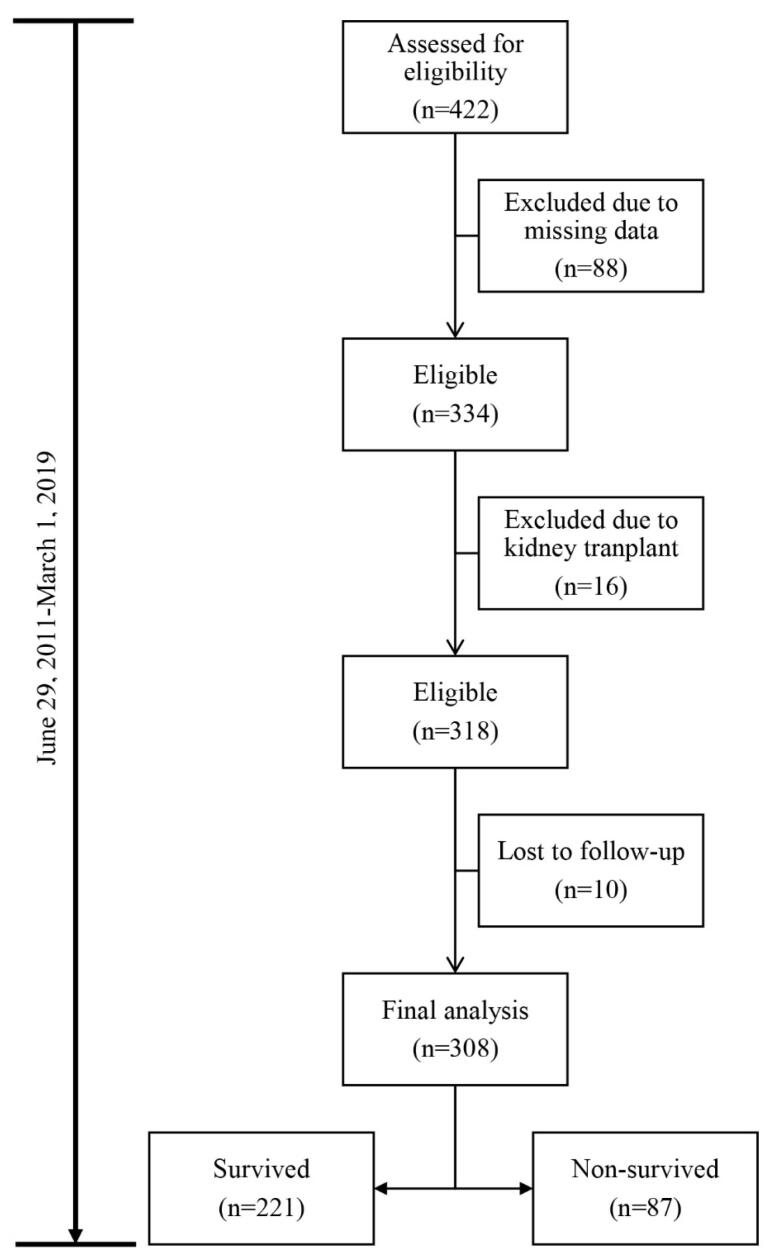
Data Structure of the Adult Male Diabetic Hemodialysis Patients Utilized in the Analyses

 Out of the 422 adult MDHD patients assessed for eligibility, 114 (27%) patients did not meet the inclusion criteria. Hence, the analyses were restricted to 308 adult MDHD patients (Figure 1). With the mean ( ± SD) age of 64.2 (12.9) (range: 27‒94) years and the body mass index (BMI) of 24.7 (2.5) (range: 14–36) kg/m^2^, the empirical mortality rate was 28.2%. The details of the baseline demographic characteristics as well as the findings of the clinical and laboratory tests are shown in Table 1.

**Table 1:** Characteristics of the Adult Male Diabetic Hemodialysis Patients in the Elastic Net Penalized Cox-Adjusted Regression Analysis

**Factors**		**Non-survivors**	**Survivors**
		**Mean (±SD)**	**Mean (±SD)**
Age (y)		63.9 (21.8)	65.0 (31.2)
Pre-dialysis weight (kg)		70.0 (10.2)	65.8 (9.9)
Post-dialysis weight (kg)		86.1 (10.0)	64.4 (9.9)
Dry weight (kg)		67.8 (11.1)	64.1 (10.2)
FBS (mg/dL)		119.1 (55.9)	121.8 (55.5)
Sodium (mEq/L)		138.8 (3.9)	138.8 (4.8)
Calcium (mg/dL)		8.6 (0.8)	8.5 (0.8)
Potassium (mEq/L)		5.0 (0.7)	5.1 (0.8)
Phosphate (mg/dL)		4.9 (1.1)	5.1 (0.8)
DBP (mm Hg)		78.9 (8.6)	77.8 (9.4)
SBP (mm Hg)		134.7 (17.3)	131.8 (17.5)
Uric acid (mg/dL)		6.4 (1.3)	6.4 (1.4)
WBC (10^6^/μL)		14.7 (20.2)	12.3 (15.7)
Iron (μg/dL)		107.9 (112.7)	116.1 (123.3)
MCHC (g/dL)		31.1 (1.5)	30.8 (1.6)
Ferritin (μg/L)		352.4 (277.1)	318.8 (239.5)
Albumin (g/dL)		3.7 (0.5)	3.8 (0.6)
Post-dialysis serum creatinine (mg/dL)		2.8 (.12)	2.9 (1.3)
Pre-dialysis serum creatinine (mg/dL)		6.6 (2.7)	6.8 (2.9)
Pre-dialysis BUN (mg/dL)		54.7 (16.9)	55.0 (14.5)
Post-dialysis BUN (mg/dL)		18.2 (7.9)	18.0 (6.1)
Cholesterol (mg/dL)		127.8 (30.2)	127.4 (27.3)
LDL (mg/dL)		74.6 (35.9)	80.6 (64.7)
Triglyceride (mg/dL)		78.8 (16.8)	80.6 (20.8)
Adequacy of dialysis (Kt/V_urea_)		1.3 (0.3)	1.3 (0.4)
UF (mL)		2.1 (0.8))	1.89 (0.9)
AST (U/L)		18.3 (9.3)	17.4 (7.4)
ALT (U/L)		16.8 (11.6)	16.0 (9.0)
ALKPH (Alkaline phosphatase) (IU/L)		335.2 (260.4)	311.6 (205.8)
Dialysis duration per session (h)	≥ 4	4.0 (0.1)	4.1 (0.1)
	< 4	3.8 (0.3)	3.6 (0.5)
BMI (Body mass index) (kg/m^2^)	< 25	23.6 (1.5)	23.4 (1.5
	≥ 25	27.2 (2.3)	27.5 (2.8)
Hemoglobin (g/dL)	< 11	9.5 (1.0)	9.2 (1.2)
	11-12.5	11.6 (0.5)	11.6 (0.4)
	> 12.5	13.5 (0.9)	14.7 (1.2)
Vascular access type^a^	AVF	89 (29)	19 (6)
	CVC	132 (43)	68 (22)
Type of membrane flux^a^	Low Flux	98 (29)	37 (12)
	High Flux	132 (43)	50 (16)

AVF, Arteriovenous fistula; BUN, Blood urea nitrogen; CVC, Central venous catheter; HDL-C, High-density lipoprotein cholesterol; LDL, Low-density lipoprotein; MCHC, Mean corpuscular hemoglobin concentration; SD, Standard deviation; FBS, Fasting blood sugar; DBP, Diastolic blood pressure; SBP, Systolic blood pressure; WBC, White blood cell; UF, Ultrafiltration volume. ^a^ Data are expressed as number (%).

**Figure 2:**
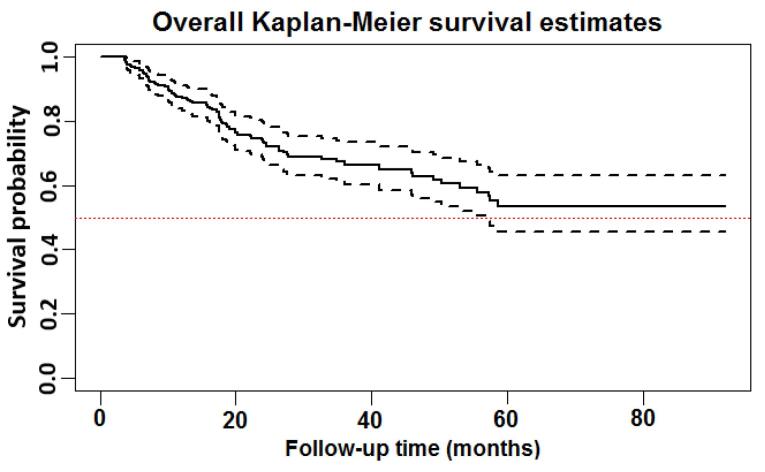
Overall Non-parametric Kaplan-Meier (KM) Survival Estimate for the Male Diabetic Hemodialysis Population (solid line) and its corresponding 95% Confidence Interval (dashed line). The total analysis time at risk and under observation was 8487 days

**Figure 3:**
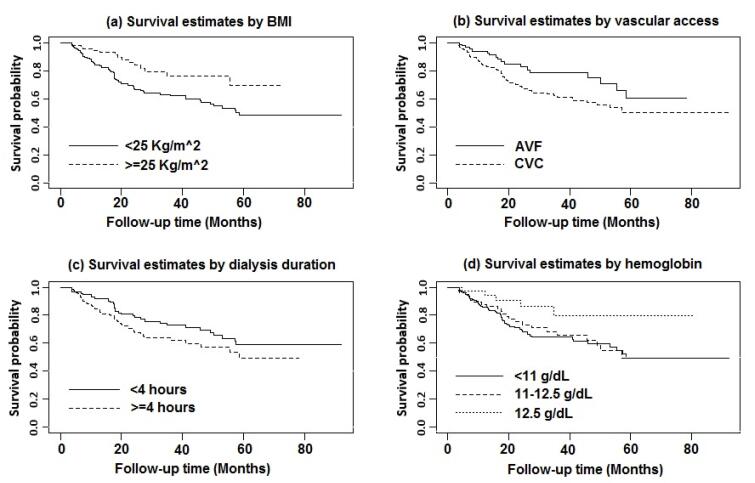
(a) BMI: < 25 kg/m^2^ (solid line) and ≥ 25 kg/m^2^ (dashed line) [Cox regression-based test: LR Chi-squared statistics = 8.68, *P* = 0.003]; (b) Initial vascular access type: AVF (solid line) and CVC (dashed line) [Cox regression-based test: LR Chi-squared statistics = 5.67, *P* = 0.017]; (c) Dialysis duration per session (hour): < 4 hour (solid line) and ≥ 4 hour (dashed line) [Cox regression-based test: LR Chi-squared statistics = 3.31, *P* = 0.069]; (d) Hemoglobin: < 11 g/dL (solid line), 11-12.5 g/dL (dashed line) and > 12.5 g/dL (dotted line) [Cox regression-based test: LR Chi-squared statistics = 6.90, *P* = 0.032]

 The KM curves for the survival function are presented in Figures 2 and 3. The curves detail the time to death in this study. The x-axes represent the time elapsed (in months) from the start (4 months after the first HD) and the y-axes are the survival probabilities. By definition, the median survival is the time at which half of the patients have experienced the desired event (i.e. death). When survival probability exceeds 50% at the longest survival time points, the median survival time cannot be calculated. Hence, in this situation, the restricted mean survival time can be used as an alternative to the median survival time.^24,27^ Using the KM plot (Figure 2), the median survival time remained undefined as more than 50% of patients were still alive. Therefore, the restricted mean survival time was calculated to be 61.5 months (95% CI: 56.3‒66.6). In addition, as Figure 2 shows, 11.4% of the patients experienced the death event by the end of the 1^st^ year, while 16.9% of them died from that point to the end of the 8-year period of dialysis data collection. The estimated overall one-, two-, three-, and eight-year survival rates (95% CI) in the MDHD patients were 87.6% (83.2‒90.9%), 74.4% (68.4‒79.4%), 67.2% (60.3‒73.1%), and 53.9% (50.0‒62.0%), respectively.

 The results of the non-parametric log-rank test showed that the MDHD patients with BMI ≥ 25 kg/m^2^ (*χ*^2^ = 8.68, *P* = 0.003), hemoglobin (Hb) level > 12.5 g/dL (*χ*^2^ = 6.90, *P* = 0.032), arteriovenous fistula (AVF) as vascular access (*χ*^2^ = 5.67, *P* = 0.017), and dialysis duration less than four hours per session (*χ*^2^ = 3.31, *P* = 0.069) were associated with higher levels of OS compared with the other patients (Figure 3).

###  Results of the Elastic Net Penalized Cox Regression 

 As a machine learning method, the EN-Cox regression model was used to model the time-to-death data. This penalized model was fitted using a combination of optimized 𝜆 values for the LASSO (*w*= 1) and Ridge (*w*= 0) regressions. The weighting (*w*) and regularization (𝜆) parameters were optimized by averaging over five repetitions of five-fold cross-validation to minimize the mean-squared error (MSE) (*w*_optimal _= 0.6 and 𝜆_optimal _= 0.0448) (Figure S1). The EN-Cox regression model retained 14 (out of 35) candidate predictors of death. The estimated shrunken coefficients for all the retained factors are presented in Table S1. These estimated model parameters may be interpreted in the same way as unpenalized regression parameters, whereby higher values indicate a greater magnitude of effect. Using the EN-Cox model, the highest magnitude effects were the initial vascular access type (coefficient = 0.52096), dialysis duration per session (coefficient = 0.49019), Hb (coefficient = -0.19175), and BMI (coefficient = -0.16474), respectively. The predictors selected by the EN-Cox model were then entered into an unpenalized Cox PH regression model to determine a baseline for comparison during the approximation of the model. The backward elimination stepwise procedure was utilized to reduce the baseline model to a parsimonious one.

 The results of the reduced EN-Cox PH model as well as the hazard ratios (HRs) (95% CI) of death are presented in Table 2. The MDHD dataset did not demonstrate any violation of the PH assumption according to the Schoenfeld residuals (all *P* values > 0.1). The *P* value of the global analysis to test the PH assumption was 0.766. Hence, it was possible to use the analysis of the EN-Cox PH model (Table 2). Moreover, the graphical evaluation of the fit of the EN-Cox model using Cox-Snell residuals is shown in Figure S2 and confirms its good performance.

**Table 2:** Hazard Ratios (95% CIs) for Time to Death in Adult Male Diabetic Hemodialysis Patients Using the Univariate and Reduced Penalized Elastic Net Cox-Adjusted Regression Model

**Factors**		**Univariable Cox regression**		**Multiple Elastic Net Cox Regression **		
		**HR (95% CI)**	***P*** ** Value**	**HR (95% CI)**	***P*** ** Value**	PH Assumption Test*
BMI (kg/m^2^)	≥ 25	1 (Reference)	—	1 (Reference)	-	—
	< 25	2.10 (1.24‒3.79)	0.006	2.75 (1.53‒4.96)	< 0.001	0.239
UF (mL/kg/h) per one-unit increase		0.75 (0.54‒0.99)	0.044	0.75 (0.57‒1.02)	0.067	0.178
Dialysis length (h)	< 4	1 (Reference)	—	1 (Reference)	—	—
	≥ 4	1.50 (0.96‒2.32)	0.072	2.95 (1.77‒4.88)	< 0.001	0.589
Vascular access	AVF	1 (Reference)	—	1 (Reference)	—	—
	CVC	1.80 (1.08‒2.99)	0.024	2.60 (1.51‒4.60)	< 0.001	0.675
SBP (mm Hg) per one-unit decrease		1.02 (1.01‒1.03)	0.046	1.02 (1.01‒1.03)	0.003	0.478
Potassium (mEq/L) per one-unit increase		1.25 (0.92‒1.68)	0.150	1.35 (0.99‒1.81)	0.059	0.253
Hemoglobin (g/dL)	> 12.5	1 (Reference)	—	1 (Reference)	—	—
	11-12.5	2.60 (0.99‒6.83)	0.052	2.99 (1.13‒7.93)	0.028	0.643
	< 11	2.90 (1.15‒7.16)	0.024	2.95 (1.18‒7.56)	0.021	0.634
LDL (mg/dL) per one-unit increase		1.002 (0.99‒1.006)	0.236	1.003 (0.99‒1.007)	0.165	0.312
HDL-C (mg/dL) per one-unit decrease		1.02 (0.99‒1.04)	0.110	1.02 (1.01‒1.05)	0.022	0.731

AVF, Arteriovenous fistula; BMI, Body mass index; CI, Confidence interval; CVC, Central venous catheter; df, Degree of freedom; HDL-C, High-density lipoprotein cholesterol; HR, Hazard ratio; LDL, Low-density lipoprotein; SBP, Systolic blood pressure; UF, Ultrafiltration volume. *Notes:* The *P* value of ≤ 0.05 is considered significant. *P value to test the proportional hazards (PH) assumption based on Schoenfeld residuals. The *P* value for global test to check the PH assumption is equal to 0.827 (chi-squared statistic = 6.45, df = 10). Therefore, the PH assumption held for all covariates in the multiple Cox-adjusted elastic net regression model.

 The regression coefficients estimated by the reduced EN-Cox model can be interpreted as the average value of each predictor’s effect on the OS rate over time. BMI < 25 kg/m^2^ (HR = 2.75, 95% CI: 1.53‒4.96, *P* < 0.001), dialysis duration ≥ 4 hour/session (HR = 2.95, 95% CI: 1.77‒4.88, *P* < 0.001), an increase in systolic blood pressure (SBP) (HR = 1.02, 95% CI: 1.01‒1.03, *P* = 0.003), hemoglobin (Hb) level < 11 g/dL (HR = 2.95, 95% CI: 1.18‒7.56, *P* = 0.021) and 11 ≤ Hb ≤ 12.5 g/dL (HR = 2.99, 95% CI: 1.13‒7.93, *P* = 0.028), and higher levels of serum high-density lipoprotein cholesterol (HDL-C) (HR = 1.02, 95% CI: 1.01‒1.03, *P* = 0.022) were associated with lower OS time in the MDHD patients. Furthermore, MDHD patients with AVF had the highest OS rate compared to those who underwent dialysis with central venous catheter (CVC) (HR = 2.60, 95% CI: 1.51‒4.60, *P* < 0.001) (Table 2).

## Discussion

 To the best of our knowledge, this was the first time that the EN-Cox regression model, as a flexible machine learning approach, was utilized to obtain a sparse regression model for predicting OS in MDHD patients. One of the major advantages of the EN-Cox regression model over the other penalization approaches is that it provides the researcher with a wide range of regression models by varying the regularization parameter (*w*) over the interval [0, 1]. In the present study, the five-fold cross-validation technique was used to select the optimal regularization parameters.

 According to the machine learning analyses in the current study, several risk factors such as duration of dialysis per session, BMI, Hb level, HDL-C, and CVC as vascular access were associated with the OS of the MDHD patients. The UF rate is a function of the amount of fluid removed during each HD session. In general, a higher ultrafiltration rate is associated with worse outcomes such as a shorter survival time and more rapid loss of residual kidney function among ESRD patients receiving HD therapy.^28^ In addition, better appetite and nutrition conditions as well as higher interdialytic weight gains lead to a higher sodium and water intake (volume overload). This can cause patients to receive a higher UF during HD with a relatively fixed duration to reduce the excess volume.^29^ It is well documented that rapid fluid removal in each session is linked to higher mortality.^29,30^ In contrast to previous studies, it was found in the current study that each unit increase in the UF volume from 1.83 mL/kg/h would decrease the risk of mortality by 25% in the MDHD patients.

 Previous studies have shown that longer HD sessions ( ≥ 3.5‒4 hours) are associated with a lower mortality rate compared with a referent group receiving less than 3.5‒4 hours of treatment.^31-33^ Similarly, in a large cohort study in the United States, Kim et al reported that the HD patients with a longer dialysis treatment time had significantly more prolonged OS, especially in subgroups with a lower UF rate. It seemed that by increasing the dialysis time, the patients could better tolerate UF, the removal of uremic toxic materials increased, the episodes of intradialytic hypotension were reduced, and the control of blood pressure was improved.^29^ In contrast to the aforementioned studies, the machine learning analysis in the present study indicated that the session lengths of ≥ 4 hours were associated with a significantly higher mortality rate (almost 3-fold) compared with a referent group receiving < 4-hour treatments. Several possible reasons can explain this finding. Most previous researches have studied the association between mortality and dialysis session length independent of the dialysis adequacy indices such as Kt/V_urea_. These studies were beset by methodological shortcomings which may have led to biased results.^33-35^ According to the penalized elastic net regression model in the current study, Kt/V_urea_ was not selected as an important risk factor for death among the MDHD patients. Therefore, it was not included in the final model for adjustment. In addition, about 30% of cases with longer session lengths (≥ 240 minutes) did not achieve adequate dialysis (i.e. their Kt/V_urea_ was < 1.2). More importantly, changes in session length over time can influence the association between the mortality rate and the dialysis session length. For instance, in a national cohort of HD patients, Brunelli et alused a marginal structural analysis to adjust the time-dependent confounding association between the session length and mortality.^36^ It has been shown that causal inference in epidemiology with time-dependent covariates in conventional statistical adjustment approaches can lead to biased estimates of the causal association.^37^ Hence, it seems that the HD session length should have been entered into the elastic net Cox model as a time-dependent feature in the current study. Moreover, it should be noted that, unlike other studies, not only a new machine learning approach was used in the current study but also a specific HD population (MDHD patients) was included for analysis. In addition, while previous studies using conventional methods considered session length as a baseline value,^33,34^ the average of the repeated measures of HD session lengths in the analyses was used in the current study in order to reduce bias in the parameter estimates.

 According to the literature, HD patients with higher BMI had a more prolonged OS. This may be due to the effects of higher BMI on decreasing HD patients’ cytokines and neurohormones. In addition, these patients have better hemodynamic status.^38-40^ Moreover, the machine learning analyses of the current study demonstrated that the risk of death for MDHD patients with BMI < 25 kg/m^2^ was 2.75 times higher than for those with BMI > 25 kg/m^2^. Although high BMI is a potential risk factor for ESRD, weight loss in patients undergoing dialysis is not recommended.^41^ Furthermore, it is worth mentioning that a high fat tissue index and a high skeletal muscle mass are more predictive than BMI and are independently associated with reduced risks of all-cause mortality in dialysis patients.^10^ Two main reasons have been suggested in this regard. First, malnutrition is very common in patients undergoing HD and is a main predictor of mortality. Second, a high BMI level is strongly associated with a higher survival rate in HD patients.^42,43^

 With the initial vascular access type, the HD process can usually be performed using the two methods of AVF and CVC.^7^ Although the risk of infection is higher in CVC, in most cases, dialysis must unavoidably be initiated with it.^44,45^ Our findings revealed that using CVC as the initial vascular access increased the risk of death about 2.6 times compared with AVF. In line with the current study, a systematic review showed that the risk for all-cause mortality due to using CVC as vascular access in HD patients was 1.5 times higher than that of AVF users.^46^ However, a substantial difference in the death rate was observed based on the initial type of vascular access (CVC or AVF). The lowest mortality rate was reported among those with AVF as the initial vascular access.^47,48^

 Erythropoietin deficiency (due to a disruption in renal erythropoietin-producing cells) in ESRD leads to anemia which is associated with several morbidities, CKD progression, and higher all-cause mortality.^49,50^ A multicenter study showed that a serum Hb level of 11‒25 g/dL could prolong OS in patients undergoing HD.^18^ Similarly, the findings of the current research revealed that the risk of death in MDHD patients with a serum Hb level of < 12.5 g/dL was almost three times higher than that of other patients. It should be noted that intensive treatment of anemia especially with erythropoietin-stimulating agents to target the serum Hb levels of > 12 g/dL may disrupt cardiovascular safety.^49^ Furthermore, high levels of ferritin and hyperkalemia due to the administration of RBC could increase the risk of infection, hospitalization, and cardiovascular events.^13^

 Our results indicated that the concentration of serum potassium tended to decrease the OS of the MDHD patients since it increased the probability of death by about 35% with a one-unit increase in its amount from 5.5 mEq/L (the threshold of hyperkalemia). Moreover, it has been well documented that hyperkalemia and hypokalemia are both potentially life-threatening conditions, especially in dialysis patients, and should be corrected immediately to prevent cardiovascular events.^51,52^ Previous researches have demonstrated that while serum potassium values < 4.0 or > 5.7 mEq/L shorten the OS of HD patients, serum potassium value of 4.6‒5.3 mEq/L increases it.^51,53^ In addition, it should be noted that the longer the interdialytic interval (more than 48 hours), the greater the odds of hyperkalemia which itself is associated with the mortality of MDHD patients.^51,54^ Although the regulation of serum potassium levels is very important, there is controversy about the concentration of dialysate potassium.^53,55^

 The level of serum HDL-C, which is inversely associated with plaque formation and the accelerated progression of atherogenesis, is reduced in dialysis patients.^12,56-58^ Our findings also revealed that the risk of mortality is reduced by 20% per one-unit increase in the level of serum HDL-C. Moradi et alfound a U-shaped relationship between HDL-C and mortality in HD patients. Their analyses demonstrated that the serum HDL-C concentrations from 50 to < 60 mg/dL were beneficial for OS in HD patients.^57^ However, no successful therapeutic strategy to increase HDL-C or to reduce the progression of CKD has been presented.^58^

 The duration of the patients’ follow-up was approximately 8 years ( > 2803 days) which was another strength of the machine learning analysis in this study. The other strong points of the current research were the substantial number of patients, using HD session indices, and employing epidemiological, clinical, and laboratory factors. A weakness of the present research was that the cause of death was not recorded in the SUMS database. Moreover, it should be mentioned that since diabetic HD patients receive proper medical health care, our results can be confounded.

## Conclusion

 Our findings revealed that a longer dialysis duration in each session ( > 4 hours), anemia (serum Hb level < 12.5 g/dL), CVC as vascular access, decrease in BMI < 25 kg/m^2^ and lower HDL-C levels could shorten the OS of MDHD patients. Also, each unit reduction in SBP increased the risk of OS by 2%. Hence, it is suggested that these factors should be modified for better survival of MDHD patients and that more attention should be paid to measuring these factors in the laboratories. The EN-Cox model used in the current study provides a new insight into using the machine learning algorithm for investigating the determinants of OS in time-to-event data. Compared with conventional survival time approaches, the EN-Cox model allows researchers to include several highly correlated factors in the regression model simultaneously. Hence, it is recommended that the EN-Cox regression model is used for survival analysis in multicollinearity cases, especially when the sample size is extremely small. In addition, when the OEPCC are too few, the EN-Cox regression model can produce stable and reliable results.

## Supplementary Files

Supplementary file 1 contains Table S1 and Figures S1 and S2.

Supplementary file 2 contains EN-Cox model theory.
