# Supplementary material for: Prognostic Features for Overall Survival in Male Diabetic Patients Undergoing Hemodialysis Using Elastic Net Penalized Cox Regression; A Machine Learning Approach
Source: Arch Iran Med. 2025 Jan 1;28(1):9–17. doi: 10.34172/aim.27746 (PMC11862393; doi:10.34172/aim.27746)
Supplement: Supplementary file 1 — contains Table S1 and Figures S1 and S2. [file aim-28-9-s001.pdf]

Arch Iran Med. January 2025;28(1):9-S1

10.34172/aim.27746

http://journalaim.com

Table S1. The selected time-to-death variables and the penalized elastic net coefficients in adult male diabetic hemodialysis patients ($\alpha_{optimal}$=0.6* and $\lambda_{optimal}$=0.0448*).

Factors	Coefficient[†]
Vascular access type	0.52096
Dialysis duration per session (h)	0.49019
Hemoglobin (g/dL)	-0.19175
BMI (Kg/m^2)	-0.16474
UF (mL)	-0.09550
Potassium (mEq/L)	0.09046
Pre-dialysis weight (Kg)	-0.02506
Phosphate (mg/dL)	0.02162
SBP (mmHg)	-0.00972
HDL-C (mg/dL)	-0.00962
Dry weight (Kg)	-0.00152
DBP (mmHg)	-0.00043
LDL (mg/dL)	0.00035
ALKPH (IU/L)	-0.00002

ALKPH, Alkaline phosphatase; BMI, Body mass index; DBP, Diastolic blood pressure; HDL-C, High-density lipoprotein cholesterol; LDL, Low-density lipoprotein; MSE, Mean-squared error; SBP, Systolic blood pressure; UF, Ultrafiltration volume.

Note: $\alpha_{optimal}$ and $\lambda_{optimal}$ are selected based on the 5-fold cross-validation for which MSE is minimum.

[†]The estimated coefficients using elastic net penalized analysis sorted by magnitude from highest to lowest.

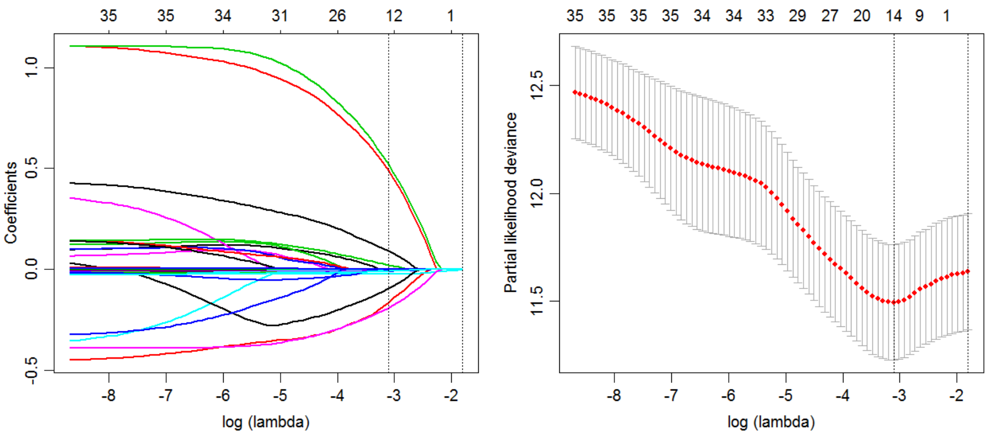

Figure S1. Left: The elastic net penalized coefficients on the male diabetic hemodialysis (MDHD) dataset are shown as a function of log (lambda). **Right:** Partial likelihood deviance of the five-fold cross-validation including lower and upper standard deviations (SDs) as a function of log (lambda) for the MDHD dataset. The dotted vertical lines demonstrate the lambda values with a minimum deviance (*log* λ = -3.1064) and the largest lambda value within one SD of the minimum deviance (*log* λ = -1.8039).

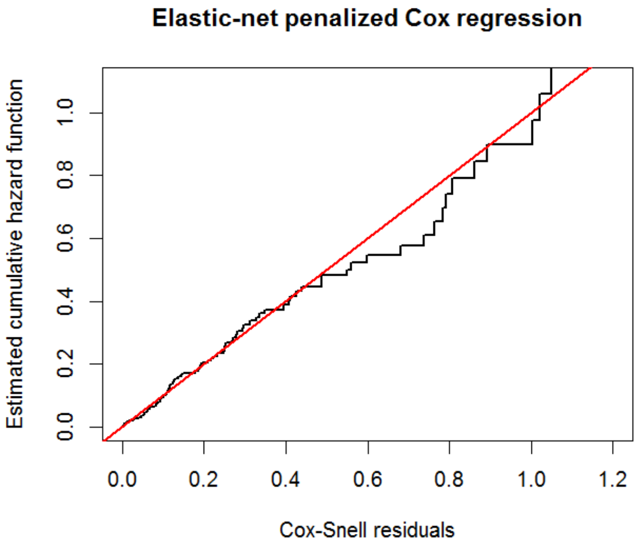

Figure S2. The graphical assessment of the fit of the elastic net penalized Cox-adjusted regression model using the Cox–Snell residual plot.
