# Supplementary material for: Prognostic Features for Overall Survival in Male Diabetic Patients Undergoing Hemodialysis Using Elastic Net Penalized Cox Regression; A Machine Learning Approach
Source: Arch Iran Med. 2025 Jan 1;28(1):9–17. doi: 10.34172/aim.27746 (PMC11862393; doi:10.34172/aim.27746)
Supplement: Supplementary file 2 — contains EN-Cox model theory. [file aim-28-9-s002.pdf]

Arch Iran Med. January 2025;28(1):9-S2

10.34172/aim.27746

http://journalaim.com

Supplementary file 2

The elastic net penalized Cox proportional hazards regression algorithm

Let we have data of the form $(\mathrm{T_j}, \boldsymbol{z}_\mathrm{j}, \varepsilon_\mathrm{j}), \mathrm{j} = 1, 2, \ldots, \mathrm{n}$ where $\mathrm{T_j}$ (the observed survival time) is a time of right-censoring if ε_j is 0 or event if ε_j is 1. As in conventional multiple regression, $\boldsymbol{z}_\mathrm{j}$ is a vector of potential covariates $(\mathrm{z_{j1}}, \mathrm{z_{j2}}, \ldots, \mathrm{z_{jS}})$. We further suppose $\mathrm{t_1}<\mathrm{t_2}<\ldots<\mathrm{t_p}$ to be the increasing sequence of unique event times, and *i(j)* shows the index of the observation failing at time $\mathrm{t_j}$. The standard Cox proportional hazards (PH) regression assumes a semi-parametric form for the hazard:

$$\mathrm{h_j(t)}=\mathrm{h_0(t)}\times e^{\sum_{\mathrm{i=1}}^{\mathrm{S}} \alpha_\mathrm{i} \mathrm{z_{ji}}}=\mathrm{h_0(t)}\times e^{\mathbf{z}_\mathrm{j}^\mathrm{T}\boldsymbol{\alpha}} \quad (1)$$

where $\mathrm{h_j(t)}$ is the hazard function for subject j at the time of t, $\mathrm{h_0(t)}$ is a baseline hazard function, and $\boldsymbol{\alpha} = (\alpha_1, \alpha_2, \ldots, \alpha_\mathrm{S})$ is a fixed, length S vector. Inference is then made through the partial likelihood function

$$\mathrm{L}(\boldsymbol{\alpha}) = \prod_{j=1}^{p} \frac{e^{\mathbf{z}_{\mathrm{i(j)}}^\mathrm{T}\boldsymbol{\alpha}}}{\sum_{\mathrm{i}\in\mathrm{R_j}} e^{\sum_{\mathrm{i=1}}^{\mathrm{S}} \alpha_\mathrm{i} \mathrm{z_{ji}}}} \quad (2).$$

Here, R_j is the set of indices, *i*, with $\mathrm{T_i}\geq\mathrm{t_j}$ (subjects at risk at the time of $\mathrm{t_j}$). By maximizing the partial *log*-likelihood function, one can estimate $\boldsymbol{\alpha}$ (1-4).

For classical cases, with many more patients than candidate covariates, the unpenalized Cox PH regression performs well. However, for small sample size data, if the number of the candidate covariates is relatively large, the number of the outcome events per candidate covariate tends to be less than expected and using conventional Cox PH regression can be misleading (5). In such cases, using penalized Cox-adjusted regressions as machine learning algorithms is the best option. These penalized methods solve this problem by adding a penalty term to the *log*-likelihood function. The penalization procedures such as elastic net and LASSO are popularly utilized for variable selection in the machine learning domain. The elastic net regression is a combination of the LASSO and ridge regressions (3, 5, 6). The elastic net penalized Cox-adjusted likelihood function is defined as

$$\mathrm{L}(\boldsymbol{\alpha})=\prod_{\mathrm{j=1}}^{\mathrm{p}} \frac{e^{\mathbf{z}_{\mathrm{i(j)}}^\mathrm{T}\boldsymbol{\alpha}}}{\sum_{\mathrm{i}\in\mathrm{R_j}} e^{\sum_{\mathrm{i=1}}^{\mathrm{S}} \alpha_\mathrm{i} \mathrm{z_{ji}}}} + \mathrm{P_{elastic\ net}}(\boldsymbol{\alpha}, w, \lambda) \quad (3)$$

where

$$\mathrm{P_{elastic\ net}}(\boldsymbol{\alpha}, w, \lambda)=\sum_{\mathrm{i=1}}^{\mathrm{S}} \lambda\left(\mathrm{w}|\alpha_\mathrm{i}|+\frac{1}{2}(1\text{-}\mathrm{w})\alpha_\mathrm{j}^2\right) \quad (4)$$

is the penalty term that penalized the estimates ($\lambda\geq0$ and $w\in[0, 1]$) (3, 6). The elastic net simplifies to simple ridge regularization when w=0, and to the LASSO regularization when w=1 (6). Furthermore, when λ=0, the penalty term is eliminated and it reduces to the ordinary partial

likelihood function. As λ increases, however, more and more model parameters shrink to 0. The trick is to specify the optimal values of the regularization parameters w and λ.

In practice, for any pre-specified w value, the optimal value of λ is determined according to the common fitness measures such as Bayes' information criterion, Akaike's information criterion, and cross validation (3). In this study, we considered the following sequence of w parameter: w=0, 0.1, 0.2, 0.3, 0.4, 0.5, 0.6, 0.7, 0.8, 0.9, and 1. For each w, we used the cross-validated partial *log*-likelihood deviance to select the appropriate regularization parameter λ so the smallest value of deviance is preferred (3, 6). The cyclical coordinate descent algorithm was utilized for maximizing the partial *log*-likelihood with the elastic net penalty (7). The "*glmnet*" R package (version 3.0-2) was used for training the elastic net penalized Cox-adjusted regression model (3).

References

1. Christensen E. Multivariate survival analysis using Cox's regression model. Hepatology. 1987;7(6):1346-58.
2. David G, Kleinbaum K. Survival analysis: a self-learning text: Springer-Verlag New York; 2016.
3. Friedman J, Hastie T, Tibshirani R. glmnet: Lasso and elastic-net regularized generalized linear models 2009 [cited 1 4]. 1-24]. Available from: https://cran.r-project.org/web/packages/glmnet/index.html.
4. Ebrahimi V, Sharifi M, Mousavi-Roknabadi RS, Sadegh R, Khademian MH, Moghadami M, et al. Predictive determinants of overall survival among re-infected COVID-19 patients using the elastic-net regularized Cox proportional hazards model: a machine-learning algorithm. 2021.
5. Peduzzi P, Concato J, Kemper E, Holford TR, Feinstein AR. A simulation study of the number of events per variable in logistic regression analysis. Journal of clinical epidemiology. 1996;49(12):1373-9.
6. Zou H, Hastie T. Regularization and variable selection via the elastic net. Journal of the royal statistical society: series B (statistical methodology). 2005;67(2):301-20. doi: https://doi.org/10.1111/j.1467-9868.2005.00503.x.
7. Simon N, Friedman J, Hastie T, Tibshirani R. Regularization paths for Cox's proportional hazards model via coordinate descent. Journal of statistical software. 2011;39(5):1. doi: https://doi.org/10.18637/jss.v039.i05.
